# A new species of *Bauhinia* L. (Caesalpinioideae, Leguminosae) from Nakhon Phanom Province, Thailand

**DOI:** 10.3897/phytokeys.26.6008

**Published:** 2013-09-09

**Authors:** Wannachai Chatan

**Affiliations:** 1 Department of Biology, Faculty of Science, Mahasarakham University, Kantharawichai District, Mahasarakham Province, 44150, Thailand

**Keywords:** *Bauhinia nakhonphanomensis*, Thailand, Leguminosae, Caesalpinioideae, new species

## Abstract

A new liana species of the subfamily Caesalpinioideae (Leguminosae), namely *Bauhinia nakhonphanomensis*, collected from the Phulangkha National Park, Nakhon Pranom Province, Thailand, is described and illustrated. It is easily recognized by the following combination of characters: tendrilled liana, entire leaves, acuminate or caudate leaf apices, oblong or elliptic floral bud, floral bud 25–35 mm long, raceme or panicle inflorescence, 10–13 mm long hypanthium, anther opening by longitudinal slits. Important comparative morphological characters with some closely related species are discussed.

## Introduction

*Bauhinia* is a large genus belonging to the subfamily Caesalpinioideae (Leguminosae). It is pantropical and consists of approx. 150–160 species (in the strict sense) and is most abundant in the neotropics (Lewis et al. 2005). The number of species reaches approx. 300 for the genus when treated in a broader sense (Larsen et al. 1984, Larsen and Larsen 1996). *Bauhinia* comprises trees, shrubs and tendrilled climbers. Its leaves are simple, entire, emarginated, bi-lobed or divided into free leaflets. Flowers are usually bisexual, with five petals and five sepals; stamens 10, 5, 3, 2 or 1 (Larsen et al. 1984). In Thailand, Larsen et al. (1984) reported 37 species occurring throughout the country. They also reported that there were six species of tendrilled climbers with entire leaves. During a plant diversity survey carried out in the years 2011-2013 in Phulangka National Park, many specimens of the leaf entire and tendrilled climbers were collected. After careful comparison with known species, it was noted that there was a climber which was quite different from any of the known taxa. Thus, this plant is described here as a new species.

## Taxonomy

### Bauhinia nakhonphanomensis

Chatan sp. nov.

urn:lsid:ipni.org:names:77131499-1

http://species-id.net/wiki/Bauhinia_nakhonphanomensis

Fig. 1

#### Type:

THAILAND, Phulangka National Park, Ban Pheang District, Nakhon Phanom Province, 17°57.087'N, 104°09.425'E, alt. 170–240 m, 28 June 2012, W. Chatan 1337 (Holotype: BK; Isotypes: MSUT).

#### Diagnosis.

*Bauhinia nakhonphanomensis* is a tendrilled liana. It differs from other closely similar species by having entire leaves, acuminate or caudate leaf apices, oblong or elliptic floral buds, floral bud 25–35 mm long, raceme or panicle inflorescences, 10–13 mm long hypanthium, and anthers opening by longitudinal slits.

#### Description.

Large tendrilled liana climbing on shrubs or trees or big rocks. Branch glabrous; small young branches straight and the old ones flattened forming “Monkey-Ladders”. *Leaves* simple; lamina ovate, 7.0–14.5 × 4.0–8.0 cm, palmately netted venation with 5 large veins near the middle and 2 short and small ones marginally; margin entire; apex acuminate or caudate; base rounded to truncate or cordate; both surfaces glabrous excepted for hairs at base of the underside of lamina; young fresh leaves pinkish and green when old. *Inflorescences* raceme or panicle, terminal or leaf axial; axes greenish and glabrous near base, reddish and covered by densely reddish hairs near apex. *Peduncles* 35–40 mm long, glabescent. *Floral buds* oblong or elliptic, 5-ridged, 25–35 × 7–9 mm, apex twisted, reddish-green when fresh and brown when dry. *Bracts* 1, insert near pedicel base, ovate or lanceolate 8–9 × 4–5 mm, reddish when fresh and brown when dry, sparse minute hairs on abaxial side, dense hairs on adaxial side. *Bracteoles* 2, insert at the pedicel apex, orbicular or broadly ovate, 10–13 × 9–10 mm, dense reddish-green hairs on adaxial side when fresh and brown when dry, dense hairs on abaxial side when fresh and brown when dry, hairs caducous. *Pedicels* 28–40 mm long, densely covered with reddish hairs when fresh, the hairs change to be brown when dry. *Hypanthium* funnel-form, 10–13 mm long, striated. *Sepals* 5, connate forming an oblong or ellipsoid shape; 5-ridged floral buds, splitting into 5 separated and recurved sepals; each sepal linear, 15–20 × 2–3 mm, abaxial side densely hairy, adaxial side sparsely hairy near apex. *Petals* 5, pinkish, spatulate, acute to obtuse apex; expanded portion 25–32 × 10–12 mm, sparsely covered by whitish hairs on both surfaces; margin entire, densely hairy on upper part and sparsely hairy on lower part; petal claw 15–22 mm long. *Stamens* 9–10; fertile stamens 3, filament 55–60 mm long, whitish to pinkish, hairy on lower part and glabrous on upper part; anther pink, sparsely hairy and 5–6 mm long and opening by longitudinal slits; sterile stamens 6–7, filament 20–24 mm long, anther 2.3–2.5 mm long, hairy and opening by longitudinal slits. *Pistil* flattened, reddish, hairy on the two ridges extending from base to the top of style; stipe 15–17 mm; ovary fusiform, 10–12 mm long; styles 12–14 mm long; stigma capitate, approx. 1 mm diameter, glabrous. *Fruit* not seen.

**Figure 1.**
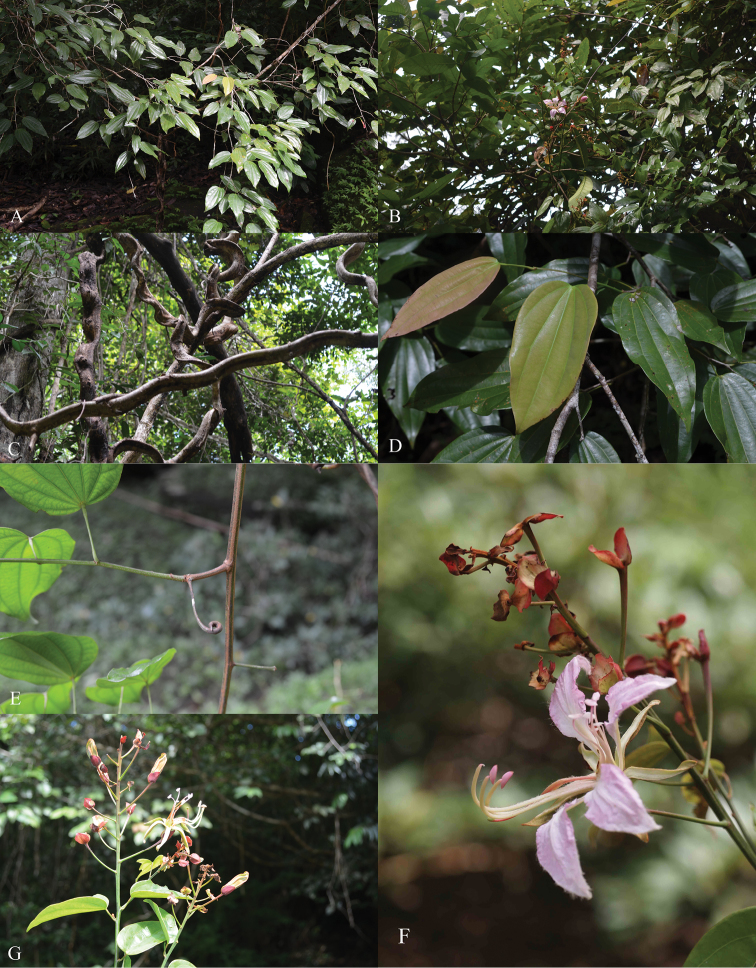
*Bauhinia nakhonphanomensis* **A** Habit **B** Habit and inflorescences **C** Large and old stems forming flattened “Monkey-Ladders” **D** Old (green) and young (pinkish) leaves **E** tendril **F** Inflorescence with many reddish bracteoles **G** Inflorescence with many reddish bracteoles and reddish-green floral buds.

#### Flowering and fruiting.

flowering April–July and fruiting unknown.

#### Distribution.

This new species is an endemic to Thailand and known from only one location at Phulangka National Park, Ban Pheang District, Nakhon Phanom Province, Thailand.

#### Ecology.

This species grows in a rocky and dense dry evergreen forest at an elevation of 170–240 m. It climbs on small to tall shrubs, trees or on big stones. Some plants grow along the river.

#### Vernacular name.

Thao Khadailing.

#### Etymology.

*Bauhinia nakhonphanomensis* is named after the type locality Nakhon Phanom Province, the northeastern Thailand.

#### Discussion.

In Thailand, *Bauhinia* species can be divided into two groups based upon their habit. The first group is comprised of trees or shrubs, while the other is tendrilled climbers. *Bauhinia nakhonphanomensis* belongs to the latter, but is clearly distinct from the other tendrilled species in having entire leaves with acuminate or caudate leaf apices, oblong or elliptic floral buds, floral bud 25–35 mm long, raceme or panicle inflorescences, 10–13 mm long hypanthium, and the anther opening by longitudinal slits. When comparing the new species to the other Thai species, it seems to closely resemble *Bauhinia concreta* Craib, *Bauhinia curtisii* Prain, *Bauhinia scandens* L., *Bauhinia strychnifolia* Craib and *Bauhinia tubicalyx* Craib based on their tendrilled climber habit, entire leaves and anther opening by longitudinal slits. *Bauhinia nakhonphanomensis* is distinct from these species by having long floral buds (i.e. 25–35 mm) and longer pedicels (i.e. 28–40 mm), while *Bauhinia concreta* Craib, *Bauhinia curtisii* Prain, *Bauhinia scandens* L., *Bauhinia strychnifolia* Craib and *Bauhinia tubicalyx* Craib have 12–15 mm floral bud lengths and shorter pedicels (2–20 mm) (Larsen et al. 1984).

When comparing this new species to the entire leaf species of *Bauhinia* in Indo-China, it can be distinguished from the other species based on hypanthium lengths. The hypanthium lengthof *Bauhinia nakhonphanomensis* is between 10–13 mm, while *Bauhinia clemensiorum* Merrill has 20–25 mm hypanthium length. Of the other entire leaf species, *Bauhinia calycima* Pierre ex Gagnep., *Bauhinia cardinalis* Pierre ex Gagnep, *Bauhinia championii* (Bentham) Bentham, *Bauhinia curtisii* Prainand, and *Bauhinia scandens* L., all have a short to very short hypanthium less than 5 mm long (Larsen et al. 1980).

*Bauhinia nakhonphanomensis* closely resembles *Bauhinia exurrens* Stapf, known only from Mt Kinabalu Malaysia (Larsen and Larsen 1996). The two species are similar to each other by having entire leaves, anthers opening by longitudinal slits, short hypanthium (approx. 10 mm), petals not recurved and long pedicels more than 25 mm. Further differences between these two species is shown in Table 1.

**Table 1.** The distinguishing features between *Bauhinia nakhonphanomensis* and *Bauhinia exurrens* Stapf.

**Characters**	***Bauhinia nakhonphanomensis***	***Bauhinia exurrens* Stapf**<br/> (from Larsen and Larsen 1996)
1. Floral bud shape	oblong or ellipsoid	clavate-ellipsoid
2. lamina nerve number	7-nerved	9–11-nerved
3. bracteole shape	Orbicular or broadly ovate	subulate
4. bracteole length	10–13 mm	2–3 mm
5. hypanthium size	10–13 mm	approx. 10 mm
6. petal color	pale pink	white?
7. petal surface	covered by whitish hairs on both surfaces	glabrous or subglabrous on both surfaces
8. petal length (including claw)	40–54 mm	approx. 20 mm
9. fertile stamen filament length	55–60 mm	15 mm
10. staminode number	6 or 7	2 or 3
11. staminode filament length	20–24	approx. 2 mm
12. stigma	globose, about 1 mm diameter	Peltate, approx. about 5 mm diameter

## Supplementary Material

XML Treatment for Bauhinia nakhonphanomensis
